# Nutritional Risk Index Improves the GRACE Score Prediction of Clinical Outcomes in Patients With Acute Coronary Syndrome Undergoing Percutaneous Coronary Intervention

**DOI:** 10.3389/fcvm.2021.773200

**Published:** 2021-12-16

**Authors:** Xiao-Teng Ma, Qiao-Yu Shao, Qiu-Xuan Li, Zhi-Qiang Yang, Kang-Ning Han, Jing Liang, Hua Shen, Xiao-Li Liu, Yu-Jie Zhou, Zhi-Jian Wang

**Affiliations:** Department of Cardiology, Beijing Anzhen Hospital, Capital Medical University, Beijing, China

**Keywords:** nutritional risk index (NRI), GRACE score, acute coronary syndrome (ACS), percutaneous coronary intervention, diabetes

## Abstract

**Background:** Malnutrition has been shown to be associated with adverse cardiovascular outcomes in many patient populations.

**Aims:** To investigate the prognostic significance of malnutrition as defined by nutritional risk index (NRI) in patients with acute coronary syndrome (ACS) undergoing percutaneous coronary intervention (PCI) and whether NRI could improve the GRACE score based prognostic models.

**Methods:** This study applied NRI among 1,718 patients with ACS undergoing PCI. Patients were divided into three nutritional risk groups according to their baseline NRI: no nutritional risk (NRI ≥ 100), mild nutritional risk (97.5 ≤ NRI <100), and moderate-to-severe nutritional risk (NRI <97.5). The primary endpoint was the composite of major adverse cardiovascular events (MACE), including all-cause death, non-fatal stroke, non-fatal myocardial infarction, or unplanned repeat revascularization.

**Results:** During a median follow-up of 927 days, 354 patients developed MACE. In the overall population, compared with normal nutritional status, malnutrition was associated with increased risk for MACE [adjusted HR for mild and moderate-to-severe nutritional risk, respectively: 1.368 (95%CI 1.004–1.871) and 1.473 (95%CI 1.064–2.041)], and NRI significantly improved the predictive ability of the GRACE score for MACE (cNRI: 0.070, *P* = 0.010; IDI: 0.005, *P* < 0.001). In the diabetes subgroup, malnutrition was associated with nearly 2-fold high adjusted risk of MACE, and the GRACE score combined with NRI appeared to have better predictive ability than that in the overall population.

**Conclusion:** Malnutrition as defined by NRI was independently associated with MACE in ACS patients who underwent PCI, especially in individuals with diabetes, and improved the predictive ability of the GRACE score based prognostic models.

## Introduction

Patients with acute coronary syndrome (ACS) are still at an unacceptably high risk of cardiovascular (CV) death and thrombotic events, even after they have undergone percutaneous coronary intervention (PCI). Comprehensive and accurate risk assessment plays an important role in making appropriate treatment decisions for these patients. The GRACE (Global Registry of Acute Coronary Events) score was a strong predictor of 6-month mortality and reinfarction after ACS (1, 2). However, some important predictors associated with poor prognosis are not included in the scoring system. Malnutrition has been proved to be associated with the development of atherosclerosis and a higher rate of CV mortality in elderly patients (3). Alternative nutritional indicators such as body mass index (BMI), serum albumin (ALB), and serum total cholesterol (TC) are predictors of survival in patients with ACS (4–6). Recently, nutritional status has been demonstrated to be a promising prognostic factor (7), and it is considered a modifiable clinical characteristic which physicians may perform interventions on to reduce the risk of adverse CV events.

The nutritional risk index (NRI) was developed as a simplified screening tool to assess nutritional status and predict clinical outcomes based on weight, height, and ALB (8). It has been reported that the malnutrition as defined by NRI was associated with the poor prognosis among patients with advanced age (9, 10), myocardial infarction (MI) (11), heart failure (HF) (12), valvular heart disease (13), atrial fibrillation (14), or chronic kidney disease (CKD) (15). So far, few studies have added nutritional status to the GRACE score for risk stratification assessment, and little is known about whether the predictive value of NRI differs among different subgroups of ACS patients. The present study aimed to evaluate the prognostic significance of nutritional status measured by NRI and the incremental predictive value of adding NRI to the GRACE score in patients with ACS undergoing PCI.

## Materials and Methods

### Study Population and Follow-Up Details

**Figure 1:**
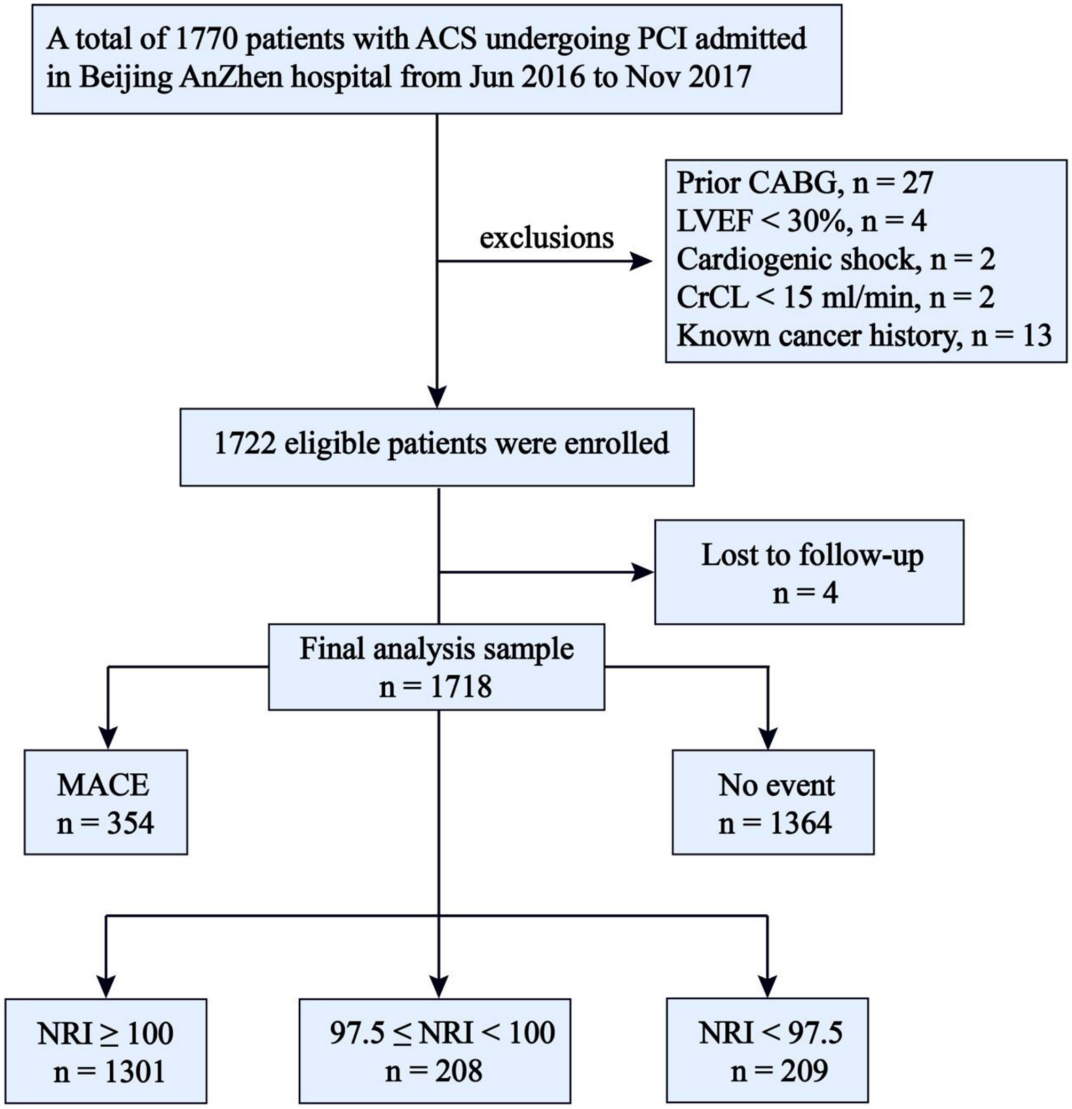
Flow chart. ACS, acute coronary syndrome; PCI, percutaneous coronary intervention; CABG, coronary artery bypass graft surgery; LVEF, left ventricular ejection fraction <30%, CrCL, creatinine clearance <15 ml/min; NRI, nutritional risk index; MACE, major adverse cardiovascular events; NRI, nutritional risk index.

The present study is derived from a single-center prospective observational cohort study (ChiCTR1800017417) which was described in detail elsewhere (16). A total of 1770 patients who underwent coronary angiography for ACS and were treated with primary or elective PCI in our CV center were consecutively and prospectively enrolled in the database from June 2016 to November 2017. The exclusion criteria of this study included patients with prior coronary artery bypass graft surgery, cardiogenic shock, left ventricular ejection fraction (LVEF) <30%, renal failure with creatinine clearance <15 ml/min, and known cancer history. Four patients were also excluded because of missing follow-up data despite at least four separate attempts to contact them. Ultimately, 1,718 patients were included in the final analysis (Figure 1). The study complied with the Declaration of the Helsinki with respect to investigation in humans, was approved by the institutional review committee of Beijing Anzhen Hospital, Capital Medical University, and conducted in accordance with the guidelines of the ethics committee at participating institutions. Written informed consent was obtained from all patients.

All patients were followed up at 1, 6, 12, 18, 24, 30, 36 months after hospital discharge. The information regarding adverse events was collected from the medical records and telephone interviews by three trained personnel blinded to the baseline characteristics. The first participant was recruited in June 2016 and the follow-up ended in December 2019.

### Clinical Outcomes

The primary endpoint was the composite of major adverse cardiovascular events (MACE), which included all-cause death, non-fatal stroke, non-fatal MI, or unplanned repeat revascularization. The stroke was defined as ischemic cerebral infarction with evidence of neurological dysfunction requiring hospitalization with clinically documented lesions on brain computed tomography or magnetic resonance imaging. MI was defined as an elevated level of cardiac troponin or creatine kinase greater than the upper limit of the normal range with either ischemic symptoms or electrocardiograph changes implicating ischemia. The presence of new pathological Q waves in ≥ 2 contiguous electrocardiogram leads was also diagnosed as MI. Within 1 week after PCI, only Q-wave MI was defined as MI. Unplanned repeat revascularization referred to any non-staged revascularization after the index PCI. Staged revascularization was defined as scheduled revascularization within 90 days after the index PCI, without a revascularization status of emergency or salvage or without treatment of a coronary artery territory which had been treated. The most severe endpoint event was selected for the primary endpoint analysis if > 1 event occurred during follow-up (death > stroke > MI > revascularization). If more than one stroke or MI or revascularization occurred, the first stroke or MI or revascularization was selected. Meanwhile, the first event was also selected for the primary endpoint analysis.

### Data Collection

Data on demographics, medical history, and medication history were collected using a standard questionnaire. The blood pressure on admission was recorded. The ALB, lipid profiles, fasting plasma glucose (FPG), glycosylated hemoglobin, high-sensitivity C-reactive protein (hs-CRP), and creatinine levels in the first fasting blood samples during the stay in the hospital, which were obtained after 12 h of fasting, were determined at the central laboratory of Beijing Anzhen Hospital. The GRACE score was calculated on admission for predicting 6-month death or MI. The symptoms of diabetes and casual plasma glucose ≥ 11.1 mmol/L, FPG ≥ 7.0 mmol/L, or 2-h plasma glucose of 75 g oral glucose tolerance test ≥ 11.1 mmol/L, and/or antidiabetic drug use were the diagnostic criteria for diabetes. Hypertension was defined as at least two blood pressure recordings greater than 140/90 mmHg, and/or use of antihypertensive drugs. Fasting TC > 5.17 mmol/L, low-density lipoprotein-cholesterol (LDL-C) > 3.36 mmol/L, triglycerides (TG) > 1.69 mmol/L, high-density lipoprotein-cholesterol (HDL-C) <1.03 mmol/L, and/or chronic use of lipid-lowering drugs were considered criteria for dyslipidemia.

### Calculation of NRI

Baseline NRI was calculated from ALB and BMI obtained on admission as previously described: NRI = 14.89 × ALB (g/dl) + 41.7 × [measured body weight (kg)/ideal body weight (kg)] [8]. The ideal body weight was calculated as follows: body height (cm)−100–{[body height (cm)−150]/4} for males, body height (cm)−100–{[body height (cm)−150]/2.5} for females (17). In accordance with prior studies, we set current body weight/ideal body weight = 1 when current body weight exceeded ideal body weight [7]. In our study, all patients were classified into three nutritional risk groups according to their baseline NRI, as defined in previous studies: normal nutrition (NRI ≥ 100), mild nutritional risk (97.5 ≤ NRI <100), and moderate-to-severe nutritional risk (NRI <97.5) (7). Due to the limitation of the sample size in this study, we did not separate a severe group since there was no patient with severe nutritional risk (NRI <83.5). GRACE score was assessed on admission for predicting 6 months death or MI.

### Statistical Analyses

Continuous variables were presented as the mean ± standard deviation or the median and interquartile range (IQR) in the case of normal or non-normal distribution and differences between two groups were examined by independent-sample *t*-test or Manne-Whitney U test correspondingly. Categorical variables were expressed as counts (percentages). The Chi-squared test or Fisher's exact test was used to analyze differences in categorical variables between groups. ANOVA or the Kruskal–Wallis H test was applied to analyze differences in continuous variables between groups. Spearman analysis was used to analyze the correlation between two continuous variables. Kaplan–Meier methods were used to derive the event rates at follow-up and to plot time-to-event curves. The NRI was analyzed in two ways: (1) as a categorical variable; and (2) as a continuous variable. Multivariate Cox proportional hazards analysis was used to estimate the hazard ratio (HR) and 95% confidence interval (CI) of NRI for MACE after adjustment for multiple confounders including other nutrition-related laboratory parameters, clinically relevant risk factors, and variables with statistical significance in the univariate analysis: lymphocyte count, neutrophil count, monocyte count, TC, hs-CRP, GRACE score, sex, BMI, current smoking, family history of CAD, hypertension, dyslipidemia, diabetes, past MI, past PCI, SYNTAX (SYNergy between percutaneous coronary intervention with TAXus and cardiac surgery) score, complete revascularization, discharged with aspirin, angiotensin converting enzyme inhibitor/angiotensin II receptor blockers (ACEI/ARBs), β-blockers, insulins, and oral antidiabetic agents. The interaction effect was tested with a likelihood ratio test, and the proportional hazard assumption was tested by demonstrating no importance of variables multiplied by time as time-dependent variables. The C-statistic, continuous net reclassification improvement (cNRI), and integrated discrimination improvement (IDI) were calculated to assess the discrimination capacity of NRI to predict CV events. All *P*-values were two-sided, and values < 0.05 were considered significant. All statistical analyses were performed using IBM SPSS Statistics version 26.0 (IBM Corporation, Chicago, IL) and R version 4.0.2 software (Vienna, Austria).

## Results

**Figure 2:**
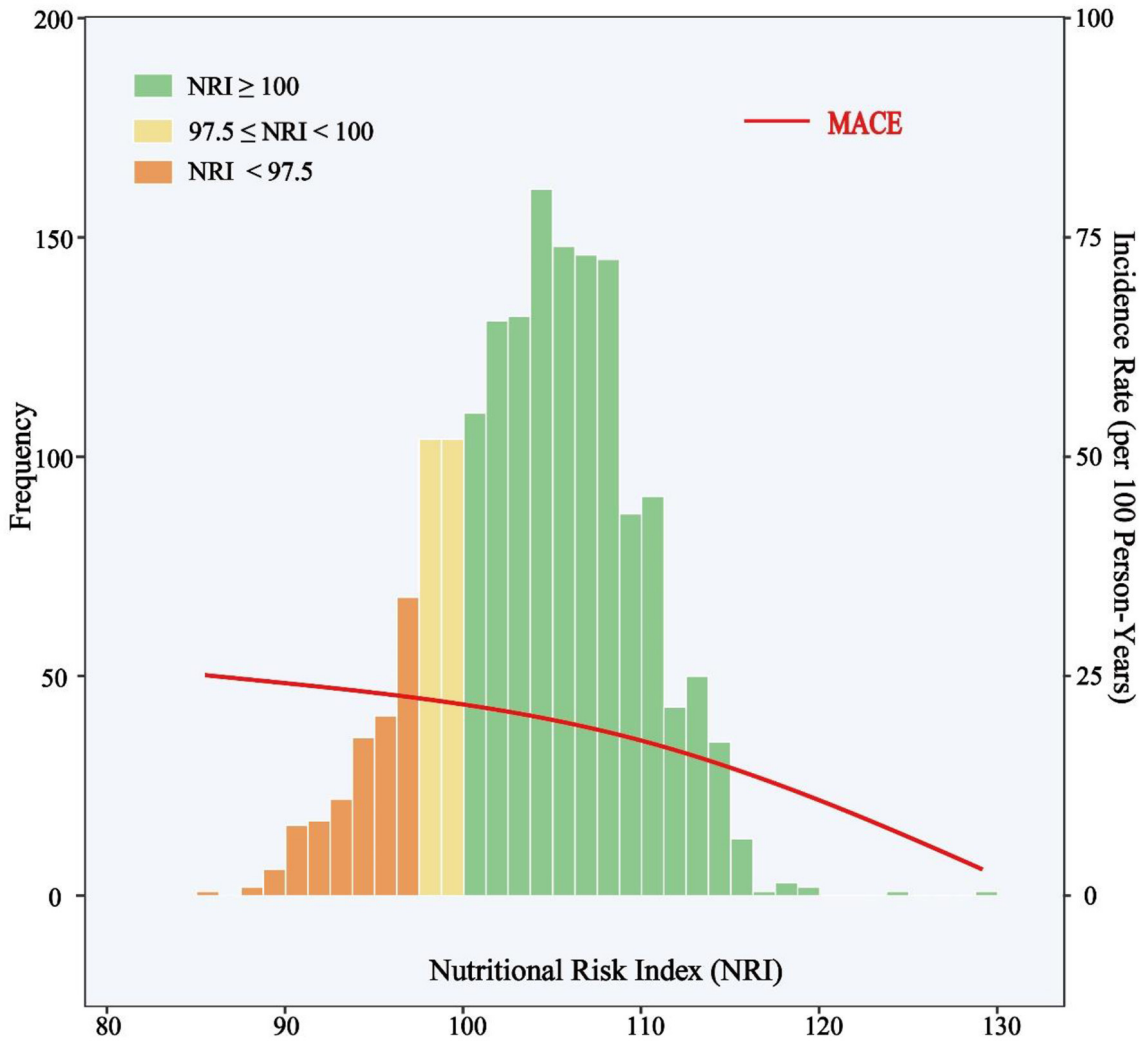
Distribution of NRI and Incidence Rate of MACE. The incidence rate curve of MACE is shown in the red line. Histograms show the population distribution of NRI. The left y-axis shows the frequency of subjects in each NRI intervals and the right y-axis shows the incidence rate (events per 100 person-years) of MACE. The x-axis shows the score of NRI by 1.5 intervals. MACE include all-cause death, non-fatal stroke, non-fatal myocardial infarction, or unplanned repeat revascularization. NRI, nutritional risk index; MACE, major adverse cardiovascular events.

The median follow-up duration was 927 days (IQR, 927 to 1,109 days), and during the follow-up period, a total of 354 patients had at least one primary endpoint event, including 239 patients from the normal nutrition group (*n* = 1301), 53 rom the mild nutritional risk group (*n* = 208), and 62 from the moderate-to-severe nutritional risk group (*n* = 209). There were 44 cases of death (37 CV deaths and seven non-CV deaths), 24 cases of non-fatal stroke, 49 cases of non-fatal MI, and 289 cases of unplanned repeat revascularization. Fifty-two patients suffered more than one primary endpoint event. The clinical outcomes according to NRI degree are shown in Table 1. The distribution of NRI and incidence rate curve of MACE across continuous NRI are shown in Figure 2. The lower the NRI, the significantly higher the incidence of MACE.

**Table 1:** Clinical outcomes according to NRI degree during follow-up.

	**All subjects**	**NRI ≥ 100**	**97.5 ≤ NRI <100**	**NRI <97.5**	***P*-value**
	**(*n* = 1,718)**	**(*n* = 1,301)**	**(*n* = 208)**	**(*n* = 209)**	
MACE-n (%)	354 (20.6)	239 (18.4)	53 (25.5)	62 (29.7)	<0.001
Death-n (%)	44 (2.6)	19 (1.5)	8 (3.8)	17 (8.1)	<0.001
Cardiovascular cause-n (%)	37 (2.2)	16 (1.2)	6 (2.9)	15 (7.2)	<0.001
Non-cardiovascular cause-n (%)	7 (0.4)	3 (0.2)	2 (1.0)	2 (1.0)	0.127
Non-fatal stroke-n (%)	24 (1.4)	12 (0.9)	5 (2.4)	7 (3.3)	0.009
Non-fatal MI-n (%)	49 (2.9)	36 (2.8)	6 (2.9)	7 (3.3)	0.895
Unplanned repeat revascularization -n (%)	289 (16.8)	206 (15.8)	40 (19.2)	43 (20.6)	0.144

*Abbreviations as in Table 2*.

At baseline, the majority of patients were male (76.7%), and the mean age was 60 years. The NRI-defined malnutrition rate was 24.3% in the total population, whereas 32.5% in the primary endpoint group, which was significantly higher than that in the event-free population. Patients with a primary endpoint event had higher heart rate, systolic blood pressure, and higher rates of family history of CAD, diabetes, previous MI and prior PCI. In terms of laboratory measurements, patients with a primary endpoint event had higher levels of creatinine, hs-CRP, neutrophil, monocyte, TC, TG, LDL-C, FPG, and glycosylated hemoglobin, but lower levels of ALB and HDL-C. As for the angiographic findings and procedural results, patients with an endpoint event had a higher SYNTAX score, a higher rate of left main or multi-vessel disease, and a lower rate of complete revascularization. Compared with those with normal nutritional status, patients with malnutrition had a higher GRACE score and higher rates of female, non-ST segment elevation MI (NSTEMI), ST segment elevation MI (STEMI), and proximal left anterior descending artery stenosis. Patients with malnutrition had higher levels of hs-CRP, neutrophil, and monocyte, but had a lower rate of complete revascularization. The baseline characteristics of the study population are shown in Table 2 and Supplementary Table 1. One-point decrease of NRI was positively correlated with hs-CRP (*r* = 0.231, *P* < 0.001), neutrophil count (*r* = 0.064, *P* = 0.008), monocyte count (*r* = 0.204, *P* < 0.001), but not significantly correlated with lymphocyte count (*r* = −0.024, *P* = 0.325).

**Table 2:** Baseline characteristics of study subjects by MACE.

**Variable**	**All subjects**	**MACE**	**No such event**	***P*-value**
	**(*n* = 1,718)**	**(*n* = 354)**	**(*n* = 1,364)**	
**NRI**	104.0 ± 5.6	103.0 ± 5.8	104.3 ± 5.5	<0.001
**NRI Degree**				<0.001
NRI ≥100-n (%)	1,301 (75.7)	239 (67.5)	1,062 (77.9)	
97.5 ≤ NRI <100-n (%)	208 (12.1)	53 (15.0)	155 (11.4)	
NRI <97.5-n (%)	209 (12.2)	62 (17.5)	147 (10.8)	
**GRACE variables**				
Age-years	60 ± 10	60 ± 11	60 ± 10	0.246
HR-bpm	69 ± 9	71 ± 10	68 ± 9	<0.001
SBP-mmHg	130 ± 16	132 ± 17	130 ± 16	0.017
Creatinine-μmol/L	70.3 (62.1–79.7)	72.0 (63.5–83.0)	69.6 (61.6–78.9)	0.003
Heart failure-n (%)	501 (29.2)	118 (33.3)	383 (28.1)	0.061
ST-segment deviation-n (%)	306 (17.8)	74 (20.9)	232 (17.0)	0.103
Elevated cardiac enzymes/markers-n (%)	443 (25.8)	97 (27.4)	346 (25.4)	0.477
Cardiac arrest-n (%)	2 (0.1)	2 (0.6)	0 (0.0)	0.057
**GRACE score**	104 ± 39	107 ± 41	103 ± 38	0.041
**GRACE risk**				0.004
Low	1,108 (64.5)	214 (60.5)	894 (65.5)	
Intermediate	287 (16.7)	52 (14.7)	235 (17.2)	
High	323 (18.8)	88 (24.9)	235 (17.2)	
**Demographics**				
Male-n (%)	1,317 (76.7)	275 (77.7)	1,042 (76.4)	0.659
Height-m	1.68 ± 0.07	1.67 ± 0.07	1.68 ± 0.07	0.322
Weight-kg	73 ± 12	72 ± 11	73 ± 12	0.075
BMI-kg/m^2^	25.7 ± 3.1	25.5 ± 3.2	25.8 ± 3.1	0.116
**Risk Factors**				
Current smokers-n (%)	759 (44.2)	168 (47.5)	591 (43.3)	0.182
Family history of CAD-n (%)	550 (32.0)	131 (37.0)	419 (30.7)	0.028
Hypertension-n (%)	1,094 (63.7)	228 (64.4)	866 (63.5)	0.797
Dyslipidemia-n (%)	1,376 (80.1)	297 (83.9)	1,079 (79.1)	0.053
Diabetes-n (%)	793 (46.2)	195 (55.1)	598 (43.8)	<0.001
Past MI-n (%)	328 (19.1)	92 (26.0)	236 (17.3)	<0.001
Past PCI-n (%)	340 (19.8)	97 (27.4)	243 (17.8)	<0.001
**Type of ACS**	
UA-n (%)	1,275 (74.2)	257 (72.6)	1,018 (74.6)	0.477
NSTEMI-n (%)	221 (12.9)	51 (14.4)	170 (12.5)	0.377
STEMI-n (%)	222 (12.9)	46 (13.0)	176 (12.9)	1.000
**Laboratory Measurements**				
ALB (g/L)	42.0 ± 3.7	41.4 ± 3.8	42.2 ± 3.6	<0.001
Lymphocyte count (x10^9^/L)	1.83 ± 0.58	1.79 ± 0.60	1.84 ± 0.58	0.130
Neutrophil count (x10^9^/L)	4.00 (3.20–4.95)	4.45 (3.56–5.41)	3.90 (3.15–4.76)	<0.001
Monocyte count (x10^9^/L)	0.36 (0.29–0.45)	0.40 (0.31–0.49)	0.35 (0.28–0.45)	<0.001
hs-CRP	1.36 (0.65–3.47)	2.22 (0.94–5.32)	1.23 (0.58–3.14)	<0.001
TC (mmol/L)	4.15 ± 0.99	4.28 ± 0.99	4.11 ± 0.99	0.005
LDL-C (mmol/L)	2.44 ± 0.81	2.55 ± 0.78	2.41 ± 0.81	0.006
HDL-C (mmol/L)	1.03 ± 0.23	0.99 ± 0.21	1.05 ± 0.24	<0.001
TG (mmol/L)	1.45 (1.01–2.06)	1.62 (1.11–2.28)	1.41 (0.98–2.01)	<0.001
FPG (mmol/L)	5.79 (5.23–6.94)	6.24 (5.45–8.02)	5.72 (5.21–6.76)	<0.001
Glycosylated hemoglobin (%)	6.1 (5.6–7.1)	6.4 (5.7–7.5)	6.0 (5.5–7.0)	<0.001
LVEF-%	65 (60–68)	62 (58–67)	65 (60–68)	<0.001
**Angiographic Findings**				
LM/multi-vessel disease-n (%)	1,458 (84.9)	323 (91.2)	1,135 (83.2)	<0.001
Proximal LAD stenosis-n (%)	862 (50.2)	193 (54.5)	669 (49.0)	0.076
SYNTAX score	21.3 ± 10.9	25.3 ± 11.0	20.2 ± 10.6	<0.001
**Procedural Results**			
DES-n (%)	1,411 (82.1)	278 (78.5)	1,133 (83.1)	0.057
BRS-n (%)	97 (5.6)	23 (6.5)	74 (5.4)	0.516
DCB-n (%)	111 (27.2)	33 (33.7)	78 (25.2)	0.128
Complete revascularization-n (%)	1,052 (61.2)	151 (42.7)	901 (66.1)	<0.001
**Medications**				
Aspirin-n (%)	1,702 (99.1)	344 (97.2)	1,358 (99.6)	<0.001
Cilostazol-n (%)	19 (1.1)	10 (2.8)	9 (0.7)	0.001
Clopidogrel-n (%)	1,576 (91.7)	320 (90.4)	1,256 (92.1)	0.358
Ticagrelor-n (%)	142 (8.3)	34 (9.6)	108 (7.9)	0.358
Statins-n (%)	1,718 (100.0)	354 (100.0)	1,364 (100.0)	NA
ACEI/ARBs-n (%)	830 (48.3)	182 (51.4)	648 (47.5)	0.211
β-blockers-n (%)	1,204 (70.1)	231 (65.3)	973 (71.3)	0.031
Any antidiabetic treatment-n (%)	572 (33.3)	158 (44.6)	414 (30.4)	<0.001
Insulin-n (%)	268 (15.6)	80 (22.6)	188 (13.8)	<0.001
Oral antidiabetic agents-n (%)	426 (24.8)	103 (29.1)	323 (23.7)	0.042
Metformin-n (%)	121 (7.0)	32 (9.0)	89 (6.5)	0.126
Alpha-glucosidase inhibitors-n (%)	281 (16.4)	65 (18.4)	281 (16.4)	0.287
Sulfonylurea-n (%)	194 (11.3)	44 (12.4)	150 (11.0)	0.506
DDP-4 inhibitors-n (%)	13 (0.8)	4 (1.1)	9 (0.7)	0.572

*MACE indicates major adverse cardiovascular events; NRI, nutritional risk index; GRACE, Global Registry of Acute Coronary Events; HR, heart rate; SBP, systolic blood pressure; BMI, body mass index; CAD, coronary artery disease; MI, myocardial infarction; PCI, percutaneous coronary; ACS, acute coronary syndrome; UA, unstable angina; NSTEMI, non-ST segment elevation myocardial infarction; STEMI, ST segment elevation myocardial infarction; ALB, albumin; hs-CRP, high-sensitivity C-reactive protein; TC, serum total cholesterol; LDL-C, low-density lipoprotein-cholesterol; HDL-C, high-density lipoprotein-cholesterol; TG, triglycerides; FPG, fasting plasma glucose; LVEF, left ventricular ejection fraction; LM, left-main artery; LAD, left anterior descending artery; SYNTAX, SYNergy between percutaneous coronary intervention with TAXus and cardiac surgery; DES, drug eluting stent; BRS, bioresorbable scaffold; DCB, drug coated balloon; ACEI, angiotensin converting enzyme inhibitor; ARB-angiotensin II receptor blocker; DDP-4, dipeptidyl peptidase 4*.

**Figure 3:**
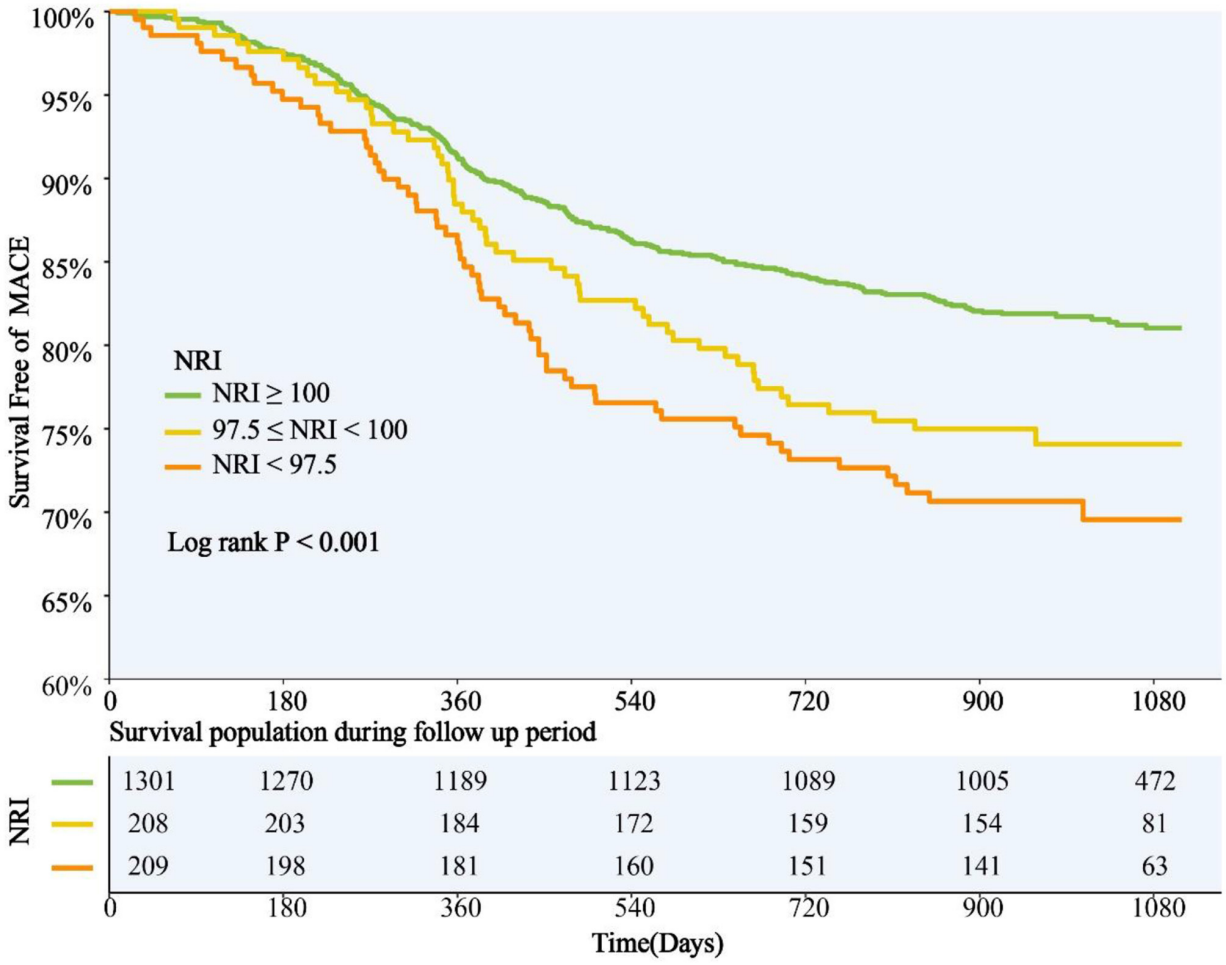
NRI Degree and Risk of MACE. Kaplan-Meier curves for MACE by the NRI degree. NRI, nutritional risk index; MACE, major adverse cardiovascular events.

**Figure 4:**
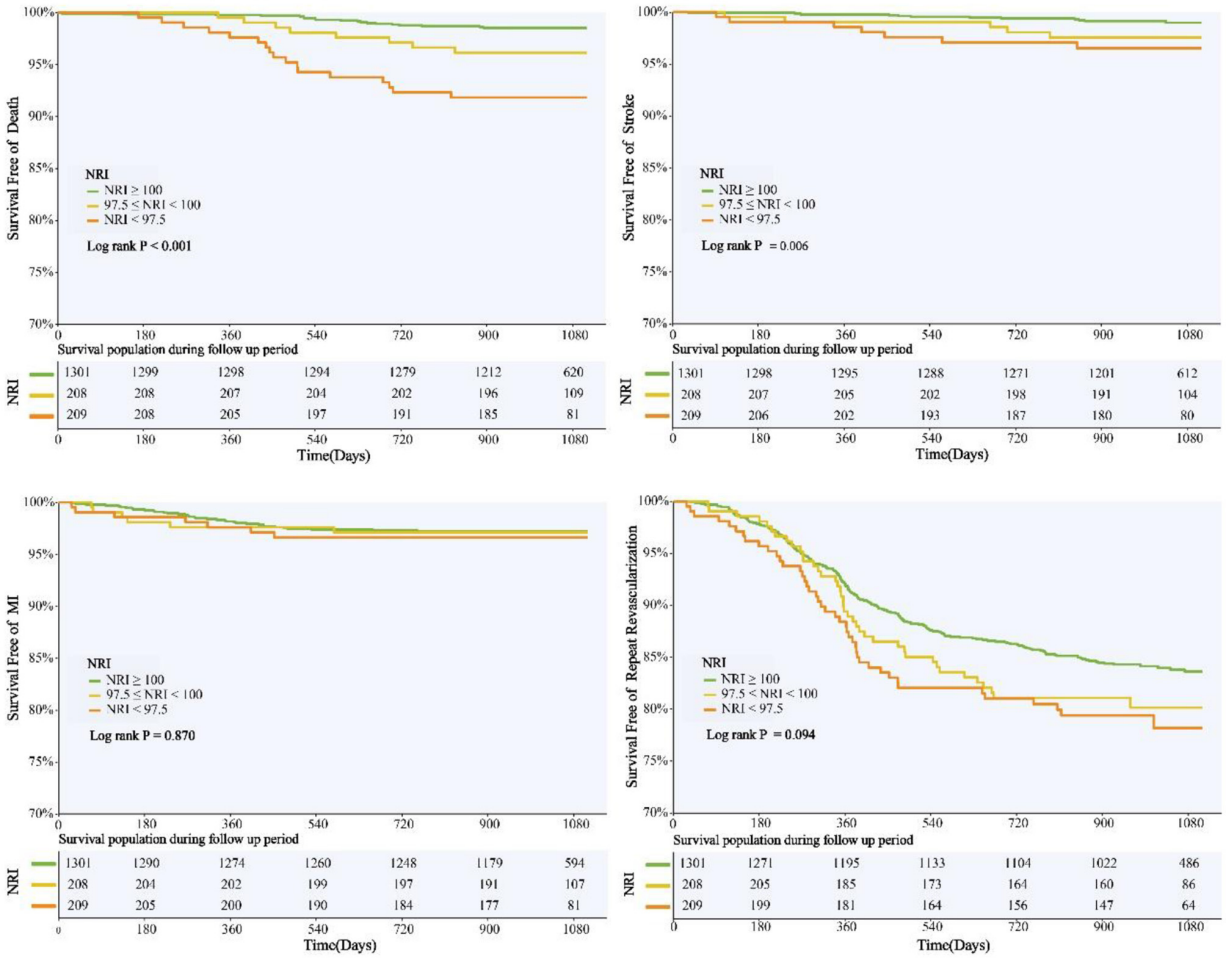
NRI Degree and Risk of Death, Stroke, MI, and Revascularization. Kaplan-Meier curves for all-cause death, non-fatal stroke, non-fatal myocardial infarction, and unplanned repeat revascularization by the NRI degree. NRI, nutritional risk index; MI, myocardial infarction.

NRI was introduced into multivariate COX regression analysis as a category variable, and after adjustment for multiple confounding factors, compared with those with normal nutritional status, patients with malnutrition had significantly higher adjusted risk of MACE in both mild and moderate-to-severe group [HR for mild and moderate-to-severe nutritional risk respectively: 1.368 (95%CI 1.004–1.871) and 1.473 (95%CI 1.064–2.041)] (Table 3). When NRI was used as a continuous variable in the multivariate Cox regression model, decreased NRI was associated with a higher risk of MACE [HR 1.026, (95%CI 1.004–1.049), *P* = 0.022] (Supplementary Table 2). Kaplan–Meier analysis revealed that patients with malnutrition showed higher incidence of the MACE (log-rank *P* < 0.001). This difference was mainly driven by the increase in death (log-rank *P* < 0.001) and stroke (log-rank *P* = 0.006), while the incidence of MI (log-rank *P* = 0.870) and repeat revascularization (log-rank *P* = 0.094) was similar between non-malnourished and malnourished patients during the follow-up. Kaplan–Meier curves of the incidence of the primary endpoint and each component of the primary endpoint for NRI are presented in Figures 3, 4.

**Table 3:** Relationship between MACE and NRI as a categorical variable in the overall population.

	**Univariate analysis**		**Multivariate analysis**	
**Variables**	**HR (95% CI)**	***P*-value**	**HR (95% CI)**	***P*-value**
NRI				
NRI ≥ 100	ref	ref	ref	ref
97.5 ≤ NRI <100	1.426 (1.059–1.920)	0.020	1.368 (1.004–1.871)	0.049
NRI <97.5	1.744 (1.319–2.306)	<0.001	1.473 (1.064–2.041)	0.020
Lymphocyte count	0.879 (0.730–1.059)	0.175	0.834 (0.682–1.020)	0.077
Neutrophil count	1.189 (1.124–1.258)	<0.001	1.117 (1.042–1.198)	0.002
Monocyte count	3.318 (1.952–5.642)	<0.001	1.446 (0.672–3.113)	0.346
TC	1.151 (1.042–1.272)	0.006	1.191 (1.070–1.326)	0.001
hs-CRP	1.032 (1.018–1.046)	<0.001	1.008 (0.988–1.027)	0.442
GRACE score	1.003 (1.000–1.005)	0.036	0.999 (0.996–1.002)	0.355
Sex	1.050 (0.818–1.349)	0.701	0.948 (0.695–1.294)	0.738
BMI	0.974 (0.940–1.008)	0.132	0.974 (0.939–1.011)	0.165
Current smoking	1.168 (0.948–1.438)	0.145	1.354 (1.056–1.736)	0.017
Family history of CAD	1.275 (1.028–1.582)	0.027	1.237 (0.992–1.543)	0.059
Hypertension	1.037 (0.834–1.289)	0.746	1.127 (0.881–1.442)	0.340
Dyslipidemia	1.346 (1.014–1.787)	0.040	1.036 (0.767–1.399)	0.819
Diabetes	1.521 (1.234–1.876)	<0.001	1.323 (0.980–1.785)	0.068
Past MI	1.530 (1.207–1.941)	<0.001	1.101 (0.833–1.457)	0.498
Past PCI	1.582 (1.252–1.998)	<0.001	1.637 (1.239–2.164)	0.001
SYNTAX score	1.036 (1.027–1.045)	<0.001	1.019 (1.009–1.030)	<0.001
Complete revascularization	0.423 (0.342–0.522)	<0.001	0.563 (0.445–0.712)	<0.001
Discharged with Aspirin	0.244 (0.130–0.457)	<0.001	0.428 (0.222–0.823)	0.011
Discharged with ACEI/ARBs	1.147 (0.931–1.412)	0.198	0.990 (0.782–1.255)	0.936
Discharged with β-blockers	0.780 (0.627–0.971)	0.026	0.716 (0.571–0.899)	0.004
Discharged with insulin	1.712 (1.335–2.197)	<0.001	1.359 (1.008–1.831)	0.044
Discharged with oral antidiabetic agents	1.293 (1.028–1.626)	0.028	0.927 (0.690–1.245)	0.615

*HR indicates hazard ratio; 95% CI, 95% confidence interval. Other abbreviations as in Table 2*.

**Figure 5:**
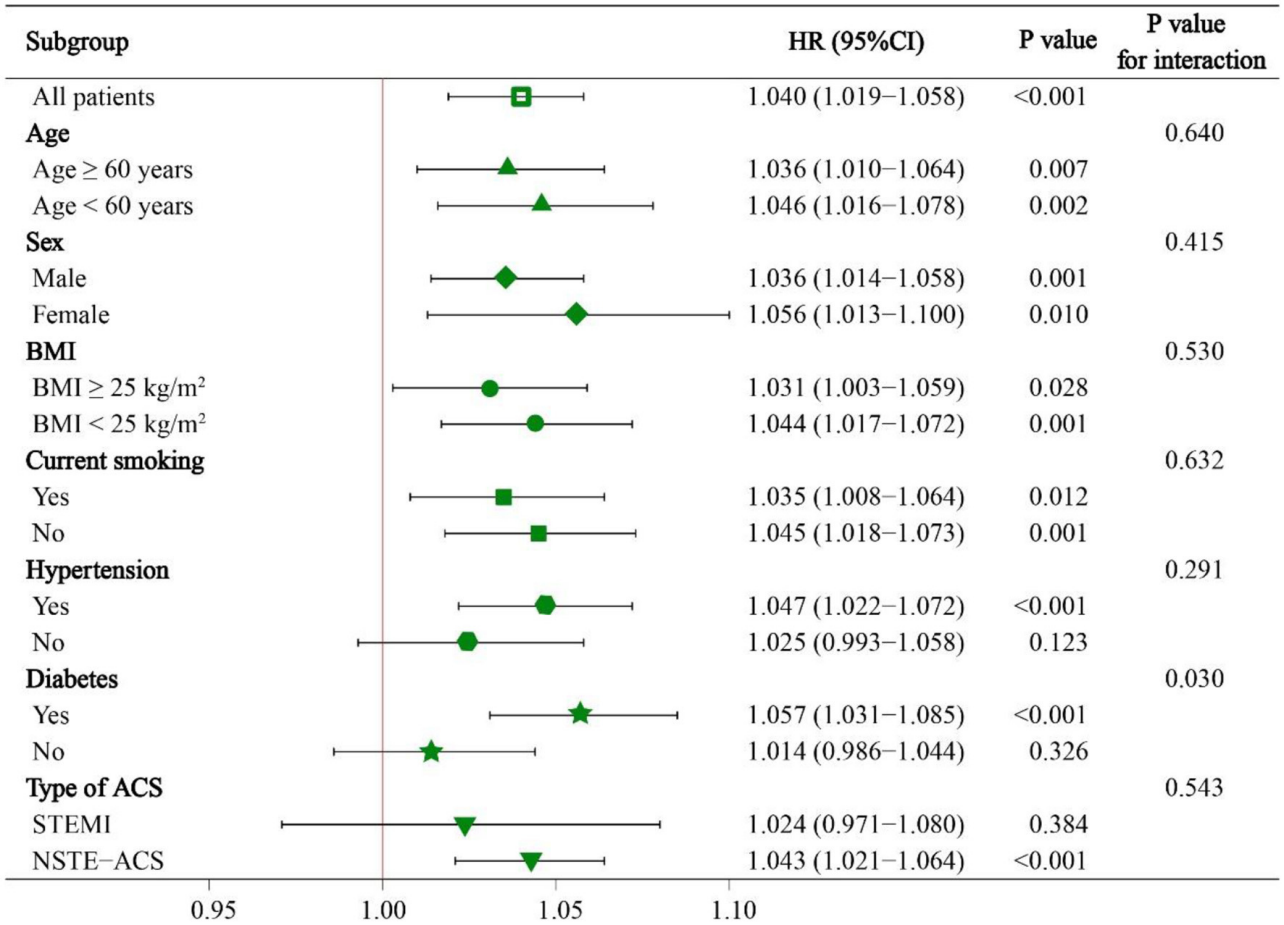
Subgroup analyses of continuous NRI for MACE. HR was evaluated by 1-point decrease of NRI. HR, hazard ratio; CI, confidence interval; BMI, body mass index; STEMI, ST-segment elevation myocardial infarction; NSTE-ACS, non-ST-segment elevation acute coronary syndrome.

Subgroup analyses were also conducted to investigate whether the predictive value of NRI was similar among patients with different demographic characteristics or comorbidities. We found a significant interaction effect between continuous NRI and diabetes subgroup (the predictive value of NRI seemed to be more prominent in patients with diabetes). However, NRI was a significant predictor of MACE regardless of age ≥ or <60 years, male or female, BMI ≥ or <25 kg/m^2^, current smoking or not, hypertension or not, STEMI or NSTE-ACS (unstable angina + NSTEMI) (all P for interaction > 0.05) (Figure 5).

Compared with the baseline GRACE score, the addition of NRI had a significant increase in C-statistic from 0.524 (95%CI 0.493–0.556) to 0.565 (95%CI 0.534–0.596) (*P* = 0.006), and significant improvement in reclassification as assessed by the cNRI (0.070, 95%CI 0.010–0.135, *P* = 0.010) and IDI (0.005, 95% CI 0.001–0.014, *P* < 0.001) (Table 4). Supplementary Table 3 shows the model performance after the addition of NRI to the baseline model in overall population.

**Table 4:** Model performance after the addition of NRI to the GRACE score in the overall population.

	**C-Statistic (95%CI)**	***P*-value**	**cNRI (95%CI)**	***P*-value**	**IDI (95%CI)**	***P*-value**
**MACE**						
GRACE	0.524 (0.493–0.556)	ref	ref	ref	ref	ref
GRACE+NRI	0.565 (0.534–0.596)	0.006	0.070 (0.010–0.135)	0.010	0.005 (0.001–0.014)	<0.001
**Death**						
GRACE	0.671 (0.577–0.764)	ref	ref	ref	ref	ref
GRACE+NRI	0.743 (0.661–0.826)	0.026	0.217 (0.037–0.400)	<0.001	0.018 (0.004–0.051)	<0.001
**Death or MI**					
GRACE	0.607 (0.540–0.673)	ref	ref	ref	ref	ref
GRACE+NRI	0.625 (0.558–0.693)	0.217	0.063 (−0.057–0.190)	0.179	0.007 (0.001–0.021)	<0.001
**Death, stroke, or MI**					
GRACE	0.633 (0.578–0.689)	ref	ref	ref	ref	ref
GRACE+NRI	0.650 (0.592–0.709)	0.165	0.088 (−0.012–0.207)	0.090	0.009 (0.002–0.026)	<0.001

*cNRI, continuous net-reclassification index; IDI, integrated discrimination improvement. Other abbreviations as in Table 2*.

We then conducted further analyses to investigate the predictive value of NRI among diabetes subjects. Compared with non-malnourished patients with diabetes, malnourished patients with diabetes had nearly 2-fold high adjusted risk of MACE [HR for mild and moderate-to-severe nutritional risk respectively: 1.601 (95%CI 1.030–2.489) and 1.977 (95%CI 1.283–3.046)] (Table 5). Compared with the baseline GRACE score, the addition of NRI had a more significant increase in C-statistic from 0.504 (95%CI 0.461–0.548) to 0.595 (95%CI 0.555–0.636) (*P* < 0.001), and more significant improvement in reclassification as assessed by the cNRI (0.176, 95%CI 0.062-0.278, *P* < 0.001) and IDI (0.022, 95% CI 0.003–0.052, *P* < 0.001) in the diabetes subgroup (Table 6). The model performance after the addition of NRI to the baseline model in diabetic population is shown in Supplementary Table 4. In addition, Supplementary Tables 5, 6 show the relationships between MACE and NRI in the overall population and diabetic population when the first event was selected for the primary endpoint analysis. NRI was an independent predictor of MACE whether the most severe event or the first event was used as the endpoint event.

**Table 5:** Relationship between MACE and NRI as a categorical variable in the diabetes subgroup.

	**Univariate analysis**		**Multivariate analysis**	
**Variables**	**HR (95% CI)**	***P*-value**		**HR (95% CI)**	***P*-value**
NRI					
NRI ≥ 100	ref	ref		ref	ref
97.5 ≤ NRI <100	1.395 (0.925–2.104)	0.112		1.601 (1.030–2.489)	0.037
NRI <97.5	2.202 (1.564–3.100)	0.000		1.977 (1.283–3.046)	0.002
Lymphocyte count	0.761 (0.590–0.980)	0.035		0.761 (0.571–1.013)	0.061
Neutrophil count	1.184 (1.090–1.287)	<0.001		1.184 (1.059–1.325)	0.003
Monocyte count	2.578 (1.311–5.070)	0.006		1.014 (0.352–2.921)	0.979
TC	1.115 (0.980–1.268)	0.099		1.213 (1.052–1.397)	0.008
hs-CRP	1.035 (1.014–1.055)	0.001		1.001 (0.972–1.031)	0.949
GRACE score	1.002 (0.999–1.005)	0.262		0.997 (0.993–0.001)	0.169
Sex	0.946 (0.691–1.294)	0.727		1.098 (0.732–1.647)	0.650
BMI	0.963 (0.919–1.008)	0.109		0.974 (0.928–0.023)	0.299
Current smoking	1.005 (0.755–1.338)	0.971		1.249 (0.887–1.758)	0.202
Family history of CAD	1.225 (0.911–1.649)	0.179		1.253 (0.916–1.713)	0.158
Hypertension	1.053 (0.777–1.428)	0.737		1.340 (0.949–1.893)	0.096
Dyslipidemia	1.313 (0.869–1.984)	0.197		0.936 (0.604–1.450)	0.766
Past MI	1.516 (1.112–2.068)	0.009		1.090 (0.753–1.579)	0.648
Past PCI	1.751 (1.304–2.351)	<0.001		1.927 (1.339–2.773)	<0.001
SYNTAX score	1.029 (1.016–1.042)	<0.001		1.016 (1.001–1.032)	0.032
Complete revascularization	0.451 (0.339–0.600)	<0.001		0.602 (0.440–0.824)	0.002
Discharged with aspirin	0.109 (0.054–0.222)	<0.001		0.292 (0.128–0.663)	0.003
Discharged with ACEI/ARBs	1.208 (0.912–1.602)	0.188		1.072 (0.778–1.476)	0.673
Discharged with β-blockers	0.776 (0.574–1.049)	0.099		0.690 (0.499–0.953)	0.024
Discharged with insulin	1.411 (1.060–1.878)	0.018		1.429 (1.046–1.951)	0.025
Discharged with oral antidiabetic agents	0.952 (0.719–1.261)	0.732		0.963 (0.710–1.306)	0.808

*Abbreviations as in Table 2*.

**Table 6:** Model performance after the addition of NRI to the GRACE score in the diabetes subgroup.

	**C-Statistic (95%CI)**	***P*-value**	**cNRI (95%CI)**	***P*-value**	**IDI (95%CI)**	***P*-value**
**MACE**						
GRACE	0.504 (0.461–0.548)	ref	ref	ref	ref	ref
GRACE+NRI	0.595 (0.555–0.636)	<0.001	0.176 (0.062–0.278)	<0.001	0.022 (0.003–0.052)	<0.001
**Death**						
GRACE	0.735 (0.586–0.884)	ref	ref	ref	ref	ref
GRACE+NRI	0.806 (0.671–0.941)	0.132	0.328 (0.004–0.566)	0.020	0.037 (0.007–0.168)	<0.001
**Death or MI**						
GRACE	0.656 (0.555–0.757)	ref	ref	ref	ref	ref
GRACE+NRI	0.698 (0.600–0.795)	0.156	0.205 (0.000–0.398)	0.050	0.018 (0.002–0.063)	0.010
**Death, stroke, or MI**						
GRACE	0.709 (0.637–0.782)	ref	ref	ref	ref	ref
GRACE+NRI	0.738 (0.664–0.812)	0.030	0.191 (0.057–0.331)	0.010	0.023 (0.004–0.062)	<0.001

*Abbreviations as in Table 2*.

## Discussion

In the present study, we noticed a significant association of NRI with CV outcomes. Compared with those with normal nutritional status, patients with malnutrition as defined by NRI had a higher risk of MACE. Even after adjustment for as many potential confounders as possible, NRI remained an independent predictor of MACE. The addition of NRI significantly improved the ability of the GRACE score to predict MACE. Intriguingly, in the diabetes subgroup, malnutrition was associated with relatively higher adjusted risk of MACE, and the GRACE score combined with NRI seemed to have better predictive ability than that in the overall population. Therefore, the present study supported the utility of NRI in predicting CV outcomes and improving the predictive ability of the model containing the GRACE score among patients with ACS.

Several reliable risk scoring models have been developed to assist clinicians in risk stratification, such as the GRACE (18), TIMI (Thrombolysis in Myocardial Infarction) (19), and CADILLAC (Controlled Abciximab and Device Investigation to Lower Late Angioplasty Complications) scores (20). Of them, the GRACE score is relatively easy to assess, and has been widely accepted as a powerful predictor of adverse CV outcomes after ACS at different time points up to 4 years (2, 18, 21). Malnutrition is common in patients with ACS and is associated with a poor prognosis regardless of GRACE score, BMI, LVEF, coronary revascularization, optimal medical treatment, and other risk factors (7). It is worth noting that variables required for nutritional status calculation are widely available, and malnutrition appears to be a potentially modifiable risk and therapeutic target.

NRI is a nutritional assessment score that includes ALB as a visceral protein element and actual weight relative to ideal weight as an anthropometric element, both of which are predictors of clinical outcomes in patients with CAD, HF, or diabetes (22, 23). However, albuminemia alone does not appear to be a reliable indicator of nutritional status, as it may be related to inflammation or hydration status rather than malnutrition (24). Hydration status is negatively correlated with ALB concentration, while positively correlated with body weight (8). The combination of both components (i.e., ALB and body weight) in the NRI counteracts the effect of hydration status on nutritional assessment. As to the NRI formula, if the current weight was higher than the ideal weight, we set weight ratio as one, which leads to a higher weighting for albumin than for weight. Otherwise, malnourished patients with overweight would not have been sensitively diagnosed. BMI is often used to define obesity, but it cannot fully reflect the nutritional status. In our study, we found that many obese patients had malnutrition and hypoalbuminemia. The study of Roubín et al. (7) showed that malnutrition was prevalent even in patients with overweight and obesity: a substantial proportion of patients with a BMI of ≥ 25 kg/m^2^ were malnourished (58% with the NRI). In the study of Sze et al. (25), one-half of heart failure patients with a BMI of ≥ 30 kg/m^2^ were malnourished as defined by Controlling Nutritional Status (CONUT) scores (another good indicator for nutritional status). These two studies suggest that malnutrition in obese people mainly manifested in low serum albumin levels, which has been shown to reflect active systematic inflammation (26–28). As we known, obesity is associated with active systemic inflammation (29). Of note, inflammation has been shown to reduce serum albumin through several possible mechanisms, including downregulation of synthesis, increased catabolism, and increased vascular permeability (30, 31). These may explain why many obese patients in our study have malnutrition and hypoalbuminemia.

Nutritional status is affected by many factors, and malnourished patients often have complex clinical conditions. In this study, NRI remained strongly associated with MACE after adjustment for multiple potential confounders, such as clinical variables, coronary revascularization, and optimal medical treatment. The two components of NRI are widely used and easily collected in the clinical practice. Therefore, NRI could be considered as a feasible and convenient tool to help predict CV outcomes. In fact, NRI was originally used to assess the nutritional status of elderly patients, who are more likely to experience unconscious weight loss (8). Although our study included patients of all ages, we also conducted an age subgroup analysis. We found no difference in the predictive value of NRI for MACE between the younger and older groups, which was consistent with the results of previous studies (15, 32).

Previous studies supported NRI-defined malnutrition as a reliable predictor of adverse CV events in many patient groups, such as patients with acute or chronic HF (12, 33, 34), patients undergoing aortic valve replacement (13, 35), and patients with other systemic diseases (14, 15, 36). Furthermore, several other studies showed that in patients with stable CAD or ACS, lower NRI levels (the lower the NRI levels, the greater the nutritional risk) were associated with in-hospital and long-term adverse CV events after PCI (11, 32, 37–39); however, these studies did not specifically investigate the prognostic value of malnutrition in ACS patients with diabetes. Since a high prevalence of diabetes-related complications and comorbidities may further impair nutritional status (40). Compared with those without diabetes, people with diabetes are more likely to suffer from malnutrition due to the diabetes itself, injuries, medications, and other factors affecting metabolism (41), which suggests that malnutrition may contribute to a higher risk of adverse CV events in ACS patients with diabetes vs. without diabetes.

Malnutrition is a complex pathological condition, and it is difficult to explain how malnutrition affects CV outcomes in patients with ACS from the results of this observational study. We believe that one of the potential mechanisms is malnutrition-inflammation-atherosclerosis (MIA) syndrome (42). ACS is the result of atherosclerotic plaque rupture causing by the chronic inflammatory response. Meanwhile, patients with diabetes are more likely to have higher levels of inflammatory markers such as C-reactive protein (43, 44), which may increase the burden of atherosclerosis (45). Thus, when malnutrition is present in patients with ACS and diabetes, hypoalbuminemia may be the result of the combination of malnutrition and inflammation. Malnutrition may be driven by inflammatory cytokines and is characterized by chronic inflammation with an increase in insulin resistance, reduction of appetite, production of catabolic cytokines, and muscle catabolism (46). While increased insulin resistance may in turn inhibit the entry of nutrients into cells and accelerate atherosclerosis (46). Another underlying mechanism of poor prognosis due to malnutrition is protein-energy malnutrition (PEM). PEM refers to a persistent state of inadequate food and nutrient intake, leading to changes in body weight, composition, and functioning (47). The association of PEM with poor prognosis in patients with acute MI, acute ischemic stroke, and HF has been demonstrated (48–50). One study showed that pre-existing PEM impaired the body's healing capability after injury, resulting in devastating clinical outcomes among patients with acute MI (51). We consider that for patients with ACS and diabetes, those who are undernourished may typically have lower cardiac and systemic muscle and nutrient reserves and may suffer more severe myocardial damage due to weaker baseline cardiac function and limited capacity to repair.

Previous studies have identified several potential risk factors beyond the GRACE score predicting model which enhance the predictive power for CV events after ACS, such as B-type natriuretic peptide (52), neutrophil count (53), and 2-h post-load glucose (54). The combination of NRI and the GRACE score produced a stronger predictive value, which improved the ability of model discrimination and risk reclassification. Our findings suggest that clinicians can apply nutritional status in combination with the GRACE score to identify higher-risk patients with ACS and diabetes and take effective measures to improve their clinical outcomes. In addition, our findings support the necessity and benefits for physicians to integrate the recognition of malnutrition in the clinical practice. Malnutrition screening in patients with ACS and diabetes can identify patients at high residual risk of CV events, who may benefit from optimized secondary prevention therapy and appropriate nutritional supplements. A recent randomized controlled trial which recruited 2,088 medical inpatients at nutritional risk found that the use of individualized nutritional support during the hospital stay improved clinical outcomes, compared with the use of standard hospital food (55). The strong evidence that individualized nutritional support can improve the prognosis of patients with HF is worth learning (56). The benefits of eating plans adjusted by the patient's preference, food fortifications, and oral nutritional supplements have been proved (57, 58). Dietary counseling and educational interventions after discharge should also be provided in the outpatient clinic. Hence, a rehabilitation unit needs physicians to cooperate with dietitians, nurses, care workers, and other professionals involved in the caring process.

This is a single-center observational study with the subsequent limitation to its nature. First, we only assessed the relationship between NRI at admission and CV outcomes and did not focus on changes in nutritional status during the follow-up. Second, the threshold of malnutrition defined by NRI is vague, and different studies set different grading criteria. Hence, there is no authoritative grading reference at present. Third, due to a limited sample size, the range of NRI was relatively small, which may affect the estimation of the relationship between NRI and CV outcomes. Fourth, all patients in this study were Chinese, so these results should be interpreted with caution and generalized to other ethnic groups since dissimilar metabolic levels exist among different races. Fifth, CKD and albuminuria might affect albumin in the blood, and glomerular hyperfiltration is one of important features in diabetic patients. In terms of kidney function, we could only obtain serum creatinine levels from the original cohort. In addition, markers of kidney injury such as albuminuria, abnormal urinary sediment, histological abnormalities, and imaging abnormalities were not routinely detected at our CV center. Therefore, we could not make an accurate diagnosis of CKD and further analyze the effect of CKD on NRI. Albuminuria was only detected qualitatively rather than quantitatively in most patients, so we could not accurately analyze the effect of albuminuria on NRI. Sixth, liver diseases might affect the clinical outcomes, or be associated with hypoalbuminemia; however, it was not taken into account in our study since indicators of liver function and information about liver diseases were not collected in the original cohort. To be clear, deferred PCI for patients with significant liver dysfunction is usually considered at our CV center unless the life-threatening conditions, so few patients with significant liver dysfunction were included in our study. Seventh, malnutrition may influence CV outcomes by promoting inflammation. Therefore, we include hs-CRP, lymphocytes, neutrophils, and monocytes into the analysis. Unfortunately, proinflammatory cytokines were not routinely measured at our CV center, so we could not analyze the correlation between proinflammatory cytokines and malnutrition or prognosis.

## Conclusion

Malnutrition as defined by NRI was independently and strongly associated with a higher risk of MACE in ACS patients who underwent PCI, especially in individuals with diabetes. NRI also improved the predictive ability of the GRACE score based prognostic models. Clinical trials are needed to determine whether improving nutritional status can improve CV outcomes in these patients.

## Data Availability Statement

The original contributions presented in the study are included in the article/Supplementary Material, further inquiries can be directed to the corresponding author.

## Ethics Statement

The studies involving human participants were reviewed and approved by the Institutional Review Committee of Beijing Anzhen Hospital, Capital Medical University. The patients/participants provided their written informed consent to participate in this study.

## Author Contributions

X-TM and Q-YS wrote the first draft of the manuscript. X-TM also made important contributions to the revision of the manuscript. X-TM, Q-YS, Q-XL, Z-QY, K-NH, JL, HS, X-LL, Y-JZ, and Z-JW were involved in the conception and design of the study and the collection, analysis, interpretation of the data, and reviewed the final manuscript. All authors read and approved the final manuscript.

## Funding

Project funded by National Key Research and Development Program of China (2017YFC0908800, Y-JZ), Beijing Municipal Administration of Hospitals' Mission plan (SML20180601, Y-JZ), China Postdoctoral Science Foundation (2021M692253, X-TM), Beijing Postdoctoral Research Foundation (2021-ZZ-023, X-TM), Beijing Municipal Health Commission (Jing 19-15, HS).

## Conflict of Interest

The authors declare that the research was conducted in the absence of any commercial or financial relationships that could be construed as a potential conflict of interest. The reviewer YC declared a shared affiliation, with the authors X-TM, Q-YS, Q-XL, Z-QY, K-NH, JL, HS, X-LL, Y-JZ, and Z-JW to the handling editor at the time of the review.

## Publisher's Note

All claims expressed in this article are solely those of the authors and do not necessarily represent those of their affiliated organizations, or those of the publisher, the editors and the reviewers. Any product that may be evaluated in this article, or claim that may be made by its manufacturer, is not guaranteed or endorsed by the publisher.

## Abbreviations

ACEI: angiotensin converting enzyme inhibitor
ACS: acute coronary syndrome
ALB: serum albumin
ARB: angiotensin II receptor blocker
BMI: body mass index
CAD: coronary artery disease
CADILLAC: Controlled Abciximab and Device Investigation to Lower Late Angioplasty Complications
CI: confidence interval
CKD: chronic kidney disease
cNRI: continuous net reclassification improvement
CONUT: Controlling Nutritional Status
CV: cardiovascular
FPG, fasting plasma glucose, GRACE: Global Registry of Acute Coronary Events
HDL-C: high-density lipoprotein-cholesterol
HF: heart failure
HR: hazard ratio
hs-CRP: high-sensitivity C-reactive protein
IDI: integrated discrimination improvement
IQR: interquartile range
LDL-C: low-density lipoprotein-cholesterol
LVEF: left ventricular ejection fraction
MACE: major adverse cardiovascular events
MI: myocardial infarction
MIA: malnutrition inflammation-atherosclerosis
NRI: nutritional risk index
NSTEMI: non-ST segment elevation myocardial infarction
NSTE-ACS: non-ST segment elevation acute coronary syndrome
PCI: percutaneous coronary intervention
PEM: protein-energy malnutrition
STEMI: ST segment elevation myocardial infarction
SYNTAX: SYNergy between percutaneous coronary intervention with TAXus and cardiac surgery
TC: serum total cholesterol
TG: Triglycerides
TIMI: Thrombolysis in Myocardial Infarction.
