# Supplementary material for: Nutritional Risk Index Improves the GRACE Score Prediction of Clinical Outcomes in Patients With Acute Coronary Syndrome Undergoing Percutaneous Coronary Intervention
Source: Front Cardiovasc Med. 2021 Dec 16;8:773200. doi: 10.3389/fcvm.2021.773200 (PMC8716456; doi:10.3389/fcvm.2021.773200)
Supplement: Supplementary file 1 [file Table_1.docx]

**Supplementary Table 1. Baseline characteristics of study subjects by NRI degree**

| **Variable** | | **All subjects**  **(n=1718)** | | **NRI≥100**  **(n=1301)** | | **97.5≤NRI<100**  **(n=208)** | | | **NRI<97.5**  **(n=209)** | | **P value** | | |
| --- | --- | --- | --- | --- | --- | --- | --- | --- | --- | --- | --- | --- | --- |
| **NRI** | | 104.0±5.6 | | 106.4±4.0 | | 98.8±0.7 | | 94.6±2.4 | | | <0.001 | |  |
| **GRACE variables** | |  | |  | |  | |  | | |  | |  |
| Age-years | | 60±10 | | 58±10 | | 63±10 | | 65±10 | | | <0.001 | |  |
| HR-bpm | | 69±9 | | 68±9 | | 70±9 | | 71±11 | | | <0.001 | |  |
| SBP-mmHg | | 130±16 | | 131±16 | | 129±16 | | 126±16 | | | <0.001 | |  |
| Creatinine-μmol/L | | 70.3 (62.1-79.7) | | 70.8 (63.2-79.3) | | 67.2 (59.3-78.5) | | 68.2 (60.2-82.3) | | | 0.009 | |  |
| Heart failure-n (%) | | 501 (29.2) | | 319 (24.5) | | 87 (41.8) | | 95 (45.5) | | | <0.001 | |  |
| ST-segment deviation-n (%) | | 306 (17.8) | | 190 (14.6) | | 55 (26.4) | | 61 (29.2) | | | <0.001 | |  |
| Elevated cardiac enzymes/markers-n (%) | | 443 (25.8) | | 289 (22.2) | | 72 (34.6) | | 82 (39.2) | | | <0.001 | |  |
| Cardiac arrest-n (%) | | 2 (0.1) | | 2 (0.2) | | 0 (0.0) | | 0 (0.0) | | | 0.725 | |  |
| **GRACE score** | | 104±39 | | 97±35 | | 119±40 | | 129±45 | | | <0.001 | |  |
| **GRACE risk** | |  | |  | |  | |  | | | <0.001 | |  |
| Low | | 1108 (64.5) | | 921 (70.8) | | 101 (48.6) | | 86 (41.1) | | |  | |  |
| Intermediate | | 287 (16.7) | | 198 (15.2) | | 42 (20.2) | | 47 (22.5) | | |  | |  |
| High | | 323 (18.8) | | 182 (14.0) | | 65 (31.2) | | 76 (36.4) | | |  | |  |
| **Demographics** | | |  | |  | |  | | |  | |  |  |
| Male-n (%) | | 1317 (76.7) | | 1032 (79.3) | | 143 (68.8) | | 142 (67.9) | | | <0.001 | |  |
| Height-m | | 1.68±0.07 | | 1.68±0.07 | | 1.67±0.09 | | 1.66±0.08 | | | <0.001 | |  |
| Weight-kg | | 73±12 | | 74±11 | | 71±13 | | 68±12 | | | <0.001 | |  |
| BMI-kg/m^2^ | | 25.7±3.1 | | 25.9±3.0 | | 25.2±3.5 | | 24.7±3.3 | | | <0.001 | |  |
| BMI<25 kg/m^2^-n (%) | | 800 (46.6) | | 562 (43.2) | | 114 (54.8) | | 124 (59.3) | | | <0.001 | |  |
| BMI≥25 kg/m^2^-n (%) | | 918 (53.4) | | 739 (56.8) | | 94 (45.2) | | 85 (40.7) | | | <0.001 | |  |
| **Risk Factors** | | |  | |  | |  | | |  | |  |  |
| Current smokers-n (%) | | 759 (44.2) | | 594 (45.7) | | 78 (37.5) | | 87 (41.6) | | | 0.065 | |  |
| Family history of CAD-n (%) | | 550 (32.0) | | 441 (33.9) | | 60 (28.8) | | 49 (23.4) | | | 0.006 | |  |
| Hypertension-n (%) | | 1094 (63.7) | | 839 (64.5) | | 126 (60.6) | | 129 (61.7) | | | 0.454 | |  |
| Dyslipidemia-n (%) | | 1376 (80.1) | | 1039 (79.9) | | 171 (82.2) | | 166 (79.4) | | | 0.709 | |  |
| Diabetes-n (%) | | 793 (46.2) | | 583 (44.8) | | 99 (47.6) | | 111 (53.1) | | | 0.075 | |  |
| Past MI-n (%) | | 328 (19.1) | | 233 (17.9) | | 52 (25.0) | | 43 (20.6) | | | 0.046 | |  |
| Past PCI-n (%) | | 340 (19.8) | | 263 (20.2) | | 42 (20.2) | | 35 (16.7) | | | 0.499 | |  |
| **Type of ACS** |  | | | | | | | | |  | |  |  |
| UA-n (%) | | 1275 (74.2) | | 1012 (77.8) | | 136 (65.4) | | 127 (60.8) | | | <0.001 | |  |
| NSTEMI-n (%) | | 221 (12.9) | | 150 (11.5) | | 35 (16.8) | | 36 (17.2) | | | 0.014 | |  |
| STEMI-n (%) | | 222 (12.9) | | 139 (10.7) | | 37 (17.8) | | 46 (22.0) | | | <0.001 | |  |
| **Laboratory Measurements** | | | | | | |  | | |  | |  |  |
| ALB (g/L) | | 42.0±3.7 | | 43.5±2.7 | | 38.7±1.0 | | 36.0±2.0 | | | <0.001 | |  |
| Lymphocyte count (x10^9^/l) | | 1.83±0.58 | | 1.84±0.57 | | 1.88±0.61 | | 1.75±0.60 | | | 0.049 | |  |
| Neutrophil counts (x10^9^/L) | | 4.00 (3.20-4.95) | | 3.97 (3.20-4.85) | | 4.01 (3.18-5.10) | | 4.26 (3.22-5.49) | | | 0.024 | |  |
| Monocyte counts (x10^9^/L) | | 0.36 (0.29-0.45) | | 0.35 (0.28-0.44) | | 0.37 (0.30-0.46) | | 0.42 (0.32-0.53) | | | <0.001 | |  |
| hs-CRP | | 1.36 (0.65-3.47) | | 1.21 (0.58-2.77) | | 1.97 (0.93-5.79) | | 3.23 (1.12-8.92) | | | <0.001 | |  |
| TC (mmol/L) | | 4.15±0.99 | | 4.20±1.00 | | 4.09±0.93 | | 3.86±0.96 | | | <0.001 | |  |
| LDL-C (mmol/L) | | 2.44±0.81 | | 2.47±0.82 | | 2.43±0.77 | | 2.28±0.79 | | | 0.006 | |  |
| HDL-C (mmol/L) | | 1.03±0.23 | | 1.05±0.23 | | 1.02±0.25 | | 0.97±0.25 | | | <0.001 | |  |
| TG (mmol/L) | | 1.45 (1.01-2.06) | | 1.50 (1.04-2.15) | | 1.36 (0.97-1.93) | | 1.26 (0.92-1.71) | | | <0.001 | |  |
| FPG (mmol/L) | | 5.79 (5.23-6.94) | | 5.77 (5.22-6.88) | | 5.76 (5.24-7.02) | | 6.13 (5.25-7.25) | | | 0.054 | |  |
| Glycosylated hemoglobin (%) | | 6.1 (5.6-7.1) | | 6.0 (5.6-7.0) | | 6.2 (5.6-7.6) | | 6.3 (5.7-7.3) | | | 0.013 | |  |
| LVEF-% | | 65 (60-68) | | 65(60-68) | | 63(58-67) | | 63(57-67) | | | <0.001 | |  |
| **Angiographic Findings** |  | | | | | |  | | |  | |  |  |
| LM/multi-vessel disease-n (%) | | 1458 (84.9) | | 1094 (84.1) | | 177 (85.1) | | 187 (89.5) | | | 0.130 | |  |
| Proximal LAD stenosis-n (%) | | 862 (50.2) | | 626 (48.1) | | 119 (57.2) | | 117 (56.0) | | | 0.010 | |  |
| SYNTAX score | | 21.3±10.9 | | 20.7±10.7 | | 22.0±11.3 | | 24.0±11.7 | | | <0.001 | |  |
| **Procedural Results** | | |  | |  | |  | | |  | |  |  |
| DES-n (%) | | 1411 (82.1) | | 1064 (81.8) | | 176 (84.6) | | 171 (81.8) | | | 0.608 | |  |
| BRS-n (%) | | 97 (5.6) | | 78 (6.0) | | 12 (5.8) | | 7 (3.3) | | | 0.305 | |  |
| DCB-n (%) | | 111 (27.2) | | 85 (28.1) | | 15 (28.8) | | 11 (20.4) | | | 0.477 | |  |
| Complete revascularization-n (%) | | 1052 (61.2) | | 814 (62.6) | | 132 (63.5) | | 106 (50.7) | | | 0.004 | |  |
| **Medications** | | |  | |  | |  | | |  | |  |  |
| Aspirin-n (%) | | 1702 (99.1) | | 1294 (99.5) | | 205 (98.6) | | 203 (97.1) | | | 0.004 | |  |
| Cilostazol-n (%) | | 19 (1.1) | | 10 (0.8) | | 3 (1.4) | | 6 (2.9) | | | 0.023 | |  |
| Clopidogrel-n (%) | | 1576 (91.7) | | 1176 (90.4) | | 195 (93.8) | | 205 (98.1) | | | <0.001 | |  |
| Ticagrelor-n (%) | | 142 (8.3) | | 125 (9.6) | | 13 (6.2) | | 4 (1.9) | | | <0.001 | |  |
| Statins-n (%) | | 1718 (100.0) | | 1301 (100.0) | | 208 (100.0) | | 209 (100.0) | | | NA | |  |
| ACEI/ARBs-n (%) | | 830 (48.3) | | 607 (46.7) | | 115 (55.3) | | 108 (51.7) | | | 0.04 | |  |
| β-blockers-n (%) | | 1204 (70.1) | | 926 (71.2) | | 129 (62.0) | | 149 (71.3) | | | 0.026 | |  |
| Any antidiabetic treatment-n (%) | | 268 (15.6) | | 197 (15.1) | | 42 (20.2) | | 29 (13.9) | | | 0.135 | |  |
| Insulin-n (%) | | 121 (7.0) | | 96 (7.4) | | 8 (3.8) | | 17 (8.1) | | | 0.146 | |  |
| Oral antidiabetic agents-n (%) | | 281 (16.4) | | 191 (14.7) | | 39 (18.8) | | 51 (24.4) | | | 0.001 | |  |
| Metformin-n (%) | | 194 (11.3) | | 154 (11.8) | | 20 (9.6) | | 20 (9.6) | | | 0.452 | |  |
| Alpha-glucosidase inhibitors-n (%) | | 13 (0.8) | | 12 (0.9) | | 0 (0.0) | | 1 (0.5) | | | 0.320 | |  |
| Sulfonylurea-n (%) | | 426 (24.8) | | 317 (24.4) | | 49 (23.6) | | 60 (28.7) | | | 0.365 | |  |
| DDP-4 inhibitors-n (%) | | 572 (33.3) | | 425 (32.7) | | 71 (34.1) | | 76 (36.4) | | | 0.553 | |  |

Abbreviations as in Table 1.
