# Supplementary material for: Nutritional Risk Index Improves the GRACE Score Prediction of Clinical Outcomes in Patients With Acute Coronary Syndrome Undergoing Percutaneous Coronary Intervention
Source: Front Cardiovasc Med. 2021 Dec 16;8:773200. doi: 10.3389/fcvm.2021.773200 (PMC8716456; doi:10.3389/fcvm.2021.773200)
Supplement: Supplementary file 2 [file Table_2.docx]

**Supplementary Table 2. Relationship between MACE and NRI as a continuous variable in the overall population**

|  | **Univariate analysis** | |  | **Multivariate analysis** | | |  |
| --- | --- | --- | --- | --- | --- | --- | --- |
| **Variables** | **HR (95% CI)** | **P value** |  | **HR (95% CI)** | **P value** | |  |
| NRI^*^ | 1.039 (1.020-1.059) | <0.001 |  | 1.026 (1.004-1.049) | | 0.022 | |
| Lymphocyte count | 0.879 (0.730-1.059) | 0.175 |  | 0.833 (0.681-1.018) | | 0.075 | |
| Neutrophil count | 1.189 (1.124-1.258) | <0.001 |  | 1.113 (1.039-1.192) | | 0.002 | |
| Monocyte count | 3.318 (1.952-5.642) | <0.001 |  | 1.472 (0.685-3.164) | | 0.322 | |
| TC | 1.151 (1.042-1.272) | 0.006 |  | 1.187 (1.067-1.321) | | 0.002 | |
| hs-CRP | 1.032 (1.018-1.046) | <0.001 |  | 1.008 (0.989-1.028) | | 0.401 | |
| GRACE score | 1.003 (1.000-1.005) | 0.036 |  | 0.998 (0.995-1.001) | | 0.283 | |
| Sex | 1.050 (0.818-1.349) | 0.701 |  | 0.951 (0.696-1.299) | | 0.753 | |
| BMI | 0.974 (0.940-1.008) | 0.132 |  | 0.971 (0.936-1.007) | | 0.109 | |
| Current smoking | 1.168 (0.948-1.438) | 0.145 |  | 1.352 (1.055-1.732) | | 0.017 | |
| Family history of CAD | 1.275 (1.028-1.582) | 0.027 |  | 1.225 (0.983-1.528) | | 0.071 | |
| Hypertension | 1.037 (0.834-1.289) | 0.746 |  | 1.124 (0.879-1.438) | | 0.351 | |
| Dyslipidemia | 1.346 (1.014-1.787) | 0.040 |  | 1.047 (0.775-1.414) | | 0.765 | |
| Diabetes | 1.521 (1.234-1.876) | <0.001 |  | 1.322 (0.98-1.784) | | 0.067 | |
| Past MI | 1.530 (1.207-1.941) | <0.001 |  | 1.123 (0.851-1.482) | | 0.413 | |
| Past PCI | 1.582 (1.252-1.998) | <0.001 |  | 1.597 (1.211-2.108) | | 0.001 | |
| SYNTAX score | 1.036 (1.027-1.045) | <0.001 |  | 1.019 (1.008-1.030) | | <0.001 | |
| Complete revascularization | 0.423 (0.342-0.522) | <0.001 |  | 0.567 (0.448-0.717) | | <0.001 | |
| Discharged with aspirin | 0.244 (0.130-0.457) | <0.001 |  | 0.398 (0.208-0.762) | | 0.005 | |
| Discharged with ACEI/ARBs | 1.147 (0.931-1.412) | 0.198 |  | 1.001 (0.790-1.268) | | 0.996 | |
| Discharged with β-blockers | 0.780 (0.627-0.971) | 0.026 |  | 0.719 (0.573-0.902) | | 0.004 | |
| Discharged with insulin | 1.712 (1.335-2.197) | <0.001 |  | 1.349 (1.002-1.817) | | 0.049 | |
| Discharged with oral antidiabetic agents | 1.293 (1.028-1.626) | 0.028 |  | 0.938 (0.698-1.262) | | 0.674 | |

^*^ HR was evaluated by 1-point decrease of NRI. Abbreviations as in Table 1 and Table 2.
