# Supplementary material for: Nutritional Risk Index Improves the GRACE Score Prediction of Clinical Outcomes in Patients With Acute Coronary Syndrome Undergoing Percutaneous Coronary Intervention
Source: Front Cardiovasc Med. 2021 Dec 16;8:773200. doi: 10.3389/fcvm.2021.773200 (PMC8716456; doi:10.3389/fcvm.2021.773200)
Supplement: Supplementary file 3 [file Table_3.docx]

**Supplementary Table 3. Model performance after the addition of NRI to the** **baseline model in the overall population**

|  | **C-Statistic (95%CI)** | **P value** | **cNRI (95%CI)** | **P value** | **IDI (95%CI)** | **P value** |
| --- | --- | --- | --- | --- | --- | --- |
| **MACE** |  |  |  |  |  |  |
| Baseline Model* | 0.708 (0.670-0.745) | ref | ref | ref | ref | ref |
| Baseline Model +NRI | 0.715 (0.677-0.752) | 0.032 | 0.088 (0.009-0.147) | 0.010 | 0.006 (0.001-0.021) | 0.010 |
| **Death** |  |  |  |  |  |  |
| Baseline Model | 0.844 (0.783-0.904) | ref | ref | ref | ref | ref |
| Baseline Model +NRI | 0.854 (0.792-0.917) | 0.142 | 0.188 (-0.042-0.377) | 0.100 | 0.012 (-0.012-0.076) | 0.299 |
| **Death or MI** | |  |  |  |  |  |
| Baseline Model | 0.765 (0.717-0.813) | ref | ref | ref | ref | ref |
| Baseline Model +NRI | 0.766 (0.718-0.814) | 0.453 | 0.145 (-0.019-0.286) | 0.060 | 0.005 (-0.001-0.025) | 0.139 |
| **Death, stroke, or MI** | |  |  |  |  |  |
| Baseline Model | 0.760 (0.716-0.804) | ref | ref | ref | ref | ref |
| Baseline Model +NRI | 0.760 (0.716-0.805) | 0.518 | 0.124 (-0.035-0.227) | 0.090 | 0.005 (-0.001-0.023) | 0.060 |

*Baseline model including lymphocyte count, neutrophil count, monocyte count, TC, hs-CRP, GRACE score, Sex, BMI, current smoking, family history of CAD, hypertension, dyslipidemia, diabetes, past MI, past PCI, SYNTAX score, complete revascularization, discharged with aspirin, discharged with ACEI/ARBs, discharged with β-blockers, discharged with insulin, discharged with oral antidiabetic agents. cNRI: continuous net-reclassification index; IDI: integrated discrimination improvement. Other abbreviations as in Table 1.
