# Supplementary material for: Nutritional Risk Index Improves the GRACE Score Prediction of Clinical Outcomes in Patients With Acute Coronary Syndrome Undergoing Percutaneous Coronary Intervention
Source: Front Cardiovasc Med. 2021 Dec 16;8:773200. doi: 10.3389/fcvm.2021.773200 (PMC8716456; doi:10.3389/fcvm.2021.773200)
Supplement: Supplementary file 4 [file Table_4.docx]

**Supplementary Table 4.** **Model performance after the addition of NRI to the baseline model in the diabetes subgroup**

|  | **C-Statistic (95%CI)** | **P value** | **cNRI (95%CI)** | **P value** | **IDI (95%CI)** | **P value** |
| --- | --- | --- | --- | --- | --- | --- |
| **MACE** |  |  |  |  |  |  |
| Baseline Model | 0.702 (0.639-0.753) | ref | ref | ref | ref | ref |
| Baseline Model +NRI | 0.734 (0.687-0.782) | 0.036 | 0.122 (0.000-0.202) | 0.050 | 0.014 (0.002-0.036) | 0.010 |
| **Death** |  |  |  |  |  |  |
| Baseline Model | 0.865 (0.776-0.954) | ref | ref | ref | ref | ref |
| Baseline Model +NRI | 0.873 (0.790-0.957) | 0.295 | 0.348 (-0.225-0.554) | 0.348 | 0.027 (-0.033-0.132) | 0.338 |
| **Death or MI** | |  |  |  |  |  |
| Baseline Model | 0.843 (0.788-0.900) | ref | ref | ref | ref | ref |
| Baseline Model +NRI | 0.842 (0.782-0.903) | 0.499 | 0.150 (-0.165-0.355) | 0.428 | 0.002 (-0.004-0.047) | 0.388 |
| **Death, stroke, or MI** | |  |  |  |  |  |
| Baseline Model | 0.832 (0.784-0.879) | ref | ref | ref | ref | ref |
| Baseline Model +NRI | 0.836 (0.787-0.885) | 0.263 | 0.122 (-0.171-0.301) | 0.527 | 0.002 (-0.006-0.048) | 0.448 |

Baseline model same as Supplementary Table 3. Abbreviations as in Table 1 and Table3.
