# Supplementary material for: Nutritional Risk Index Improves the GRACE Score Prediction of Clinical Outcomes in Patients With Acute Coronary Syndrome Undergoing Percutaneous Coronary Intervention
Source: Front Cardiovasc Med. 2021 Dec 16;8:773200. doi: 10.3389/fcvm.2021.773200 (PMC8716456; doi:10.3389/fcvm.2021.773200)
Supplement: Supplementary file 5 [file Table_5.docx]

**Supplementary Table 5. Relationship between MACE and NRI as a categorical variable in the overall population (the first event as endpoint)**

|  | **Univariate analysis** | |  | | **Multivariate analysis** | | | | | | |  |  |  |
| --- | --- | --- | --- | --- | --- | --- | --- | --- | --- | --- | --- | --- | --- | --- |
| **Variables** | **HR (95% CI)** | **P value** | | | |  | | **HR (95% CI)** | | | **P value** | | | |
| NRI |  |  | |  | | |  | |  | | | |  |  |
| NRI ≥ 100 | ref | ref | |  | | | ref | | ref | | | |  |  |
| 97.5 ≤ NRI < 100 | 1.428 (1.060-1.923) | 0.019 | |  | | | 1.365 (1.001-1.865) | | | 0.049 | | | |  |
| NRI < 97.5 | 1.756 (1.328-2.321) | <0.001 | |  | | | 1.481 (1.070-2.049) | | | 0.018 | | | |  |
| Lymphocyte count | 0.873 (0.725-1.052) | 0.154 | |  | | | 0.829 (0.678-1.013) | | | 0.067 | | | |  |
| Neutrophil count | 1.190 (1.125-1.258) | <0.001 | |  | | | 1.116 (1.041-1.196) | | | 0.002 | | | |  |
| Monocyte count | 3.307 (1.944-5.625) | <0.001 | |  | | | 1.399 (0.651-3.006) | | | 0.389 | | | |  |
| TC | 1.151 (1.041-1.271) | 0.006 | |  | | | 1.193 (1.071-1.329) | | | 0.001 | | | |  |
| hs-CRP | 1.033 (1.019-1.048) | <0.001 | |  | | | 1.010 (0.991-1.030) | | | 0.316 | | | |  |
| GRACE score | 1.003 (1.000-1.005) | 0.030 | |  | | | 0.999 (0.996-1.002) | | | 0.362 | | | |  |
| Sex | 1.056 (0.822-1.356) | 0.669 | |  | | | 0.957 (0.702-1.306) | | | 0.783 | | | |  |
| BMI | 0.973 (0.939-1.007) | 0.121 | |  | | | 0.973 (0.938-1.009) | | | 0.141 | | | |  |
| Current smoking | 1.169 (0.949-1.440) | 0.143 | |  | | | 1.361 (1.061-1.744) | | | 0.015 | | | |  |
| Family history of CAD | 1.275 (1.027-1.581) | 0.028 | |  | | | 1.243 (0.997-1.551) | | | 0.054 | | | |  |
| Hypertension | 1.034 (0.832-1.286) | 0.760 | |  | | | 1.125 (0.880-1.440) | | | 0.347 | | | |  |
| Dyslipidemia | 1.335 (1.006-1.773) | 0.046 | |  | | | 1.025 (0.759-1.385) | | | 0.872 | | | |  |
| Diabetes | 1.514 (1.228-1.867) | <0.001 | |  | | | 1.313 (0.973-1.773) | | | 0.075 | | | |  |
| Past MI | 1.532 (1.208-1.942) | <0.001 | |  | | | 1.117 (0.845-1.477) | | | 0.438 | | | |  |
| Past PCI | 1.578 (1.249-1.993) | <0.001 | |  | | | 1.624 (1.229-2.147) | | | 0.001 | | | |  |
| SYNTAX score | 1.036 (1.027-1.045) | <0.001 | |  | | | 1.020 (1.009-1.031) | | | <0.001 | | | |  |
| Complete revascularization | 0.423 (0.342-0.522) | <0.001 | |  | | | 0.567 (0.448-0.717) | | | <0.001 | | | |  |
| Discharged with aspirin | 0.247 (0.132-0.463) | <0.001 | |  | | | 0.444 (0.230-0.855) | | | 0.015 | | | |  |
| Discharged with ACEI/ARBs | 1.151 (0.935-1.418) | 0.185 | |  | | | 0.994 (0.785-1.260) | | | 0.963 | | | |  |
| Discharged with β-blockers | 0.781 (0.628-0.972) | 0.027 | |  | | | 0.724 (0.577-0.909) | | | 0.005 | | | |  |
| Discharged with insulin | 1.710 (1.333-2.194) | <0.001 | |  | | | 1.361 (1.010-1.833) | | | 0.043 | | | |  |
| Discharged with oral antidiabetic agents | 1.284 (1.021-1.615) | 0.033 | |  | | | 0.923 (0.687-1.240) | | | 0.594 | | | |  |

HR indicates hazard ratio; 95% CI, 95% confidence interval. Other abbreviations as in Table 1.
