# Supplementary material for: Nutritional Risk Index Improves the GRACE Score Prediction of Clinical Outcomes in Patients With Acute Coronary Syndrome Undergoing Percutaneous Coronary Intervention
Source: Front Cardiovasc Med. 2021 Dec 16;8:773200. doi: 10.3389/fcvm.2021.773200 (PMC8716456; doi:10.3389/fcvm.2021.773200)
Supplement: Supplementary file 6 [file Table_6.docx]

**Supplementary Table 6. Relationship between MACE and NRI as a categorical variable in the diabetes subgroup (the first event as endpoint)**

|  | **Univariate analysis** | |  | | **Multivariate analysis** | | | | | | |  |  |  |
| --- | --- | --- | --- | --- | --- | --- | --- | --- | --- | --- | --- | --- | --- | --- |
| **Variables** | **HR (95% CI)** | **P value** | | | |  | | **HR (95% CI)** | | | **P value** | | | |
| NRI |  |  | |  | | |  | |  | | | |  |  |
| NRI ≥ 100 | ref | ref | |  | | | ref | | ref | | | |  |  |
| 97.5 ≤ NRI < 100 | 1.404 (0.931-2.117) | 0.106 | |  | | | 1.603 (1.031-2.491) | | | 0.036 | | | |  |
| NRI < 97.5 | 2.210 (1.570-3.112) | <0.001 | |  | | | 1.956 (1.270-3.013) | | | 0.002 | | | |  |
| Lymphocyte count | 0.759 (0.589-0.978) | 0.033 | |  | | | 0.763 (0.573-1.016) | | | 0.064 | | | |  |
| Neutrophil count | 1.188 (1.093-1.291) | <0.001 | |  | | | 1.185 (1.060-1.325) | | | 0.003 | | | |  |
| Monocyte count | 2.598 (1.324-5.100) | 0.006 | |  | | | 0.992 (0.346-2.843) | | | 0.988 | | | |  |
| TC | 1.151 (1.042-1.272) | 0.006 | |  | | | 1.204 (1.045-1.389) | | | 0.010 | | | |  |
| hs-CRP | 1.037 (1.016-1.057) | 0.037 | |  | | | 1.003 (0.974-1.034) | | | 0.824 | | | |  |
| GRACE score | 1.002 (1.000-1.005) | 0.050 | |  | | | 0.997 (0.993-1.001) | | | 0.191 | | | |  |
| Sex | 0.949 (0.694-1.299) | 0.746 | |  | | | 1.096 (0.731-1.644) | | | 0.657 | | | |  |
| BMI | 0.962 (0.919-1.008) | 0.105 | |  | | | 0.973 (0.927-1.022) | | | 0.283 | | | |  |
| Current smoking | 1.013 (0.761-1.348) | 0.932 | |  | | | 1.263 (0.897-1.779) | | | 0.180 | | | |  |
| Family history of CAD | 1.219 (0.906-1.640) | 0.190 | |  | | | 1.238 (0.905-1.694) | | | 0.182 | | | |  |
| Hypertension | 1.049 (0.774-1.421) | 0.760 | |  | | | 1.324 (0.937-1.872) | | | 0.112 | | | |  |
| Dyslipidemia | 1.316 (0.871-1.989) | 0.192 | |  | | | 0.941 (0.607-1.458) | | | 0.784 | | | |  |
| Past MI | 1.526 (1.119-2.081) | 0.008 | |  | | | 1.092 (0.755-1.580) | | | 0.640 | | | |  |
| Past PCI | 1.760 (1.311-2.363) | <0.001 | |  | | | 1.940 (1.348-2.790) | | | <0.001 | | | |  |
| SYNTAX score | 1.029 (1.016-1.042) | <0.001 | |  | | | 1.016 (1.001-1.031) | | | 0.035 | | | |  |
| Complete revascularization | 0.453 (0.341-0.602) | <0.001 | |  | | | 0.607 (0.443-0.830) | | | 0.002 | | | |  |
| Discharged with aspirin | 0.111 (0.054-0.225) | <0.001 | |  | | | 0.296 (0.130-0.673) | | | 0.004 | | | |  |
| Discharged with ACEI/ARBs | 1.211 (0.913-1.605) | 0.184 | |  | | | 1.066 (0.773-1.469) | | | 0.697 | | | |  |
| Discharged with β-blockers | 0.778 (0.576-1.052) | 0.103 | |  | | | 0.696 (0.504-0.963) | | | 0.029 | | | |  |
| Discharged with insulin | 1.416 (1.064-1.885) | 0.017 | |  | | | 1.435 (1.051-1.958) | | | 0.023 | | | |  |
| Discharged with oral antidiabetic agents | 0.946 (0.714-1.253) | 0.699 | |  | | | 0.954 (0.704-1.294) | | | 0.762 | | | |  |

HR indicates hazard ratio; 95% CI, 95% confidence interval. Other abbreviations as in Table 1.
